# 

**Published:** 1893-08-05

**Authors:** 


**OUR NEW OFFICES**
428, Strand, W.C., will henceforth be the Office of this Journal.

**Notices of Births, Deaths, and Marriages,** are inserted in this column at a charge of 2s. 6d. for an announcement not exceeding four lines.

### NOTICE TO CORRESPONDENTS.

All MS., Letters, Books for Review, and other matters intended for the Editor should be addressed The Editor, The Lodge, Porchester Square, London, W.

The Editor cannot undertake to return rejected M.S., even when accompanied by stamped directed envelope.

**Notice to Advertisers and Subscribers.**

All Advertisements, Orders for Copies of The Hospital, and Business Communications should be addressed to The Publisher, *not to the Editor*, The Hospital, 428, Strand, W.C.

The Cost of Subscription, post free, is as follows:—Per week, 2½d.; per quarter, 2s. 6d.; per half-year, 5s.; per annum, 8s. 6d. Foreign:—Per annum, 10s. 8d.; payable, in all cases, in advance.

**"Burdett's Hospital Annual," 1893.**

Fourth year of publication. 700 pp. Boards, gilt lettering. A most complete guide to British, Colonial, Indian, and American Hospitals, Dispensaries, Nursing and Convalescent Institutions, and Asylums. Price 5s. Published by The Scientific Press (Limited), 428, Strand, W.C.

**NOTICE.—The Index for Vol. XIII. (ending March 1893) is on sale at the Office of this Journal, price 2½d., post free.**

### Books Received.

George Bell and Sons.

"Mountaineering." By Claude Wilson, M.D.

Charles Griffin and Co.

"The Physiologist's Note Book." By Alex. Hill, M.D.

W. H. Allen and Co.

"Words on Existing Religions." By the Hon. Albert Canning.

Methuen and Co.
"Back to the Land." By Harold E. Moore, F.S.I.

Griffith, Lanen, and Co.
"Dust and Laurels: A Study in 19th Century Womanhood." By M. Pendered.

Bailliere, Tindall, and Cox.
"A Handbook of Obstetric and Gynæcological Nursing." By the late Fleetwood Churchill, M.D.

Henry Kempton.
"Indigestion, Gout, Corpulency, and Constipation." By Thomas Dutton, M.D. Third Edition.

Smith, Elder, and Co.

"First Aid to the Injured." By Dr. Friedrich Esmach. Translated by H.R.H. The Princess Christian.

H. K. Lewis.
"Tooth Extractions." By John Gorham, M.R.C.S.

F. V. White and Co.

"Aunt Johnnie." By John Strange Winter.

"Basil and Annette." By B. L. Farjeon.

"Housekeeping." By Mrs. Humphry.

**Periodicals and Pamphlets.**—Birmingham Medical Review, Charity Organisation Review, The "Amines" Process, Clinical Journal, Strand, and Talk, The Review of Reviews, Religious Review of Reviews, The Review of the Churches. London. The Therapist, Christian Commonwealth, Cassell's Magazine, Cornhill Magazine, Talk, London, The Therapeutic Gazette, The Indian Medico Chirurgical Review, Edinburgh Medical Missionary Society, Quarterly Paper, Reichs.' Medicinal Anzeiger, Guy's Hospital Gazette, Chicago Clinical Journal, Work and Leisure, Natural Food, The Humanitarian, Amateur Gardening, The News, Edinburgh Medical Journal.

The Religious Tract Society: The Sunday at Home, Leisure Hour, Girl's Own Paper, Boy's Own Paper.

### The Hospital Advertiser.

### Medical Vacancies and Appointments.

### APPOINTMENTS.

L. Baine, M.D., B.S.Durh., D.P.H.Camb., F.C.S., appointed Resident Physician to the Fever Hospital, Newcastle-on-Tyne. H. P. D. Benson, M.B.Edin., appointed Medical Officer for the Skenfrith District of the Monmouth Union. Frances May D. Berry, M.D.Lond., appointed Pathologist to the New Hospital for Women. T. Carr, M.B.Durh., M.R.C.S.Eng., L.S.A.Lond., appointed Medical Officer of Health to the Braintree Rural Sanitary Authority. A. H. Carter, M.D.Lond., M.R.C.S., appointed Honorary Medical Officer to the Corbett Hospital, Stourbridge. T. F. Chavasse, M.D., F.R.C.S.Edin., appointed Honorary Consulting Surgeon to the Corbett Hospital, Stourbridge. W. R. Dakin, M.D., appointed Physician to the General Lying-in Hospital. H. D'A. Ellis, L.R.C.P.Edin., M.R.C.S.Eng., appointed Honorary Surgeon to the Corbett Hospital, Stourbridge. J. J. Flinn, L.R.C.P.Edin., M.R.C.S.Eng., appointed Medical Officer for the Belmont Road Workhouse of the West Derby Union. A. M. Gossage, M.B.Oxon., appointed Medical Registrar at Westminster Hospital. H. Gurney, L.R.C.P., L.M., L.R.C.S. Edin., reappointed Medical Officer of Health for the Borough of Harwich. E. G. Hall, M.B.Lond., M.R.C.S., appointed Surgeon to the Bristol Dispensary. A. W. Hare, appointed Medical Officer for the Keyworth District of the Bingham Union. Dr. Hogarth, appointed *pro tem.* District Medical Officer for the Cheltenham Union. D. W. C. Hood, M.D.Cantab., F.R.C.P., appointed Consulting Physician to the North-West London Hospital, Kentish Town Road. R. Howden, M.B., C.M., appointed Medical Officer of Health for the Burgh of Haddington. R. K. Howat, M.B., C.M.Glas., M.R.C.S., L.R.C.P.Lond., appointed Assistant House Surgeon to the General Hospital, Wolverhampton. J. Kekwick, L.D.S.Eng., appointed House Surgeon to the Dental Hospital of London. A. B. Lyon, M.B., C.M.Aberd., appointed Medical Officer for the Macduff Sanitary District. J. F. McDougall, M.R.C.S.Eng., L.R.C.P.Edin., appointed Assistant Medical Officer of Health for the Urban Sanitary District of Walton-on-the-Hill. R. D. Muir, L.R.C.P.Lond., M.R.C.S., appointed Resident Medical Officer to Queen Charlotte's Lying-in Hospital. A. Purkiss, M.D., appointed Medical Officer for the Walston District of the Rugby Union. F. Roberts, M.B., appointed Resident Medical Officer to the Leeds Infirmary. H. H. Smith, L.R.C.P. Lond., M.R.C.S., appointed Honorary Surgeon to the Corbett Hospital, Stourbridge. W. A. Wills, M.D.Lond., M.R.C.P., M.R.C.S., appointed Assistant Physician to the Westminster Hospital. W. J. Woods, M.B., B.Ch., B.A.O.R.U.I., appointed Junior House Surgeon to the Royal Southern Hospital, Liverpool.

### VACANCIES.

The following vacancies are announced this week, the amount of salary in each case being given after the name of the institution:

**Resident Medical Officers.**

Chesterfield and North Derbyshire Hospital and Dispensary, Chesterfield. House Surgeon. Salary, £100 per annum. Assistant House Surgeon. Salary £50, with board, apartments, and laundress for each. Applications to the Secretary, by August 4th.

Liverpool Infirmary for Children. Assistant House Surgeon. Appointment for six months, board and lodging provided. Application to C. W. Carver, Honorary Secretary, by August 5th.

Victoria Infirmary of Glasgow. Superintendent and Resident Medical Officer. Applications to Francis Bisset, Secretary, 22, Carlton Place, Glasgow, by August 15th.

**Non-Resident Medical Officers.**

National Orthopædic Hospital, 234, Great Portland Street, W. Surgical Registrar. Honorarium £20 per annum. Applications to M. A. H. Tubby, F.R.C.S., at the hospital.

Portumna Union, Portumna Dispensary No. 2. Medical Officer. Salary £40 per annum, with £5 as Medical Officer of Health, registration and vaccination fees. Applications to Mr. Laurence Taylor, Honorary Secretary. Election on August 5th.

Tralee Union, Tralee Dispensary. Apothecary. Salary £20 per annum. Applications to Mr. W. Denny, Honorary Secretary. Election on August 6th.

Westminster Hospital, Broad Sanctuary, S.W. Surgeon. Must be Fellow of the Royal College of Surgeons of England. Applications with testimonials, to the Secretary, S. M. Quennel, by August 22nd.

### PASS LISTS.

**Royal College of Physicians of Edinburgh, Royal College of Surgeons of Edinburgh, and Faculty of Physicians and Surgeons of Glasgow.**

The quarterly examinations in Edinburgh for the Triple Qualification took place in July, with the following results:—

First Examination (*under the entire Examination system*).—Of

**ROYAL COLLEGE OF SURGEONS OF EDINBURGH.**

### MEDICAL VACANCIES AND APPOINTMENTS—(*continued*)

thirty-two candidates the following twenty-five passed:—J. A. Featherstone, County Durham (with honours); E. Barker, Yorkshire; D. Hennesey, County Cork; W. F. Macfarlane, Bangalore; H. W. G. J. J. Cattell, Mauritius; S. O. Hall, India; P. J. H. Mulholland, County Clare; T. A. Chalmers, Ayr; J. A. S. Cantell, Cheshire; W. Mangan, County Kerry; M. V. McKechnie, County Durham; G. H. Williams, Scarborough; P. A. Wedgwood, London; R. A. Bowen, Kerry; S. G. A. Jeffs, Lincolnshire; Ada Mary Kingsbury, Sydney; Pieter Hermanus du Plessis, Cape Colony · T. F, Elmes, Cork; R. J. Molison, Upper Holloway; H. J. M. Wyllys, Great Yarmouth; J. W. Lee, Liverpool; G. T. Harbord, Cheshire; E. B. Hicks, Yorkshire; J. S. D. Robertson, Alness; and W. A. McDonald, County Cork. Three candidates entered for the respective divisions and passed. *Under Five Years' Course.*—Of sixteen candidates, the following twelve passed: J. G. Murray. Hong Kong; J. M. Donovan, Galbally; J. Dodds, Selkirk; J. St. J. Murphy, County Cork; J. Murray, Newcastleton; Marion Balfour Marshall, Edinburgh; Elizabeth Morton Johnston, Edinburgh; T. J. T. McHattie, Antigua; Ellen Willet Stanley, Calais; Janet Dorothy Gilfillan, London; A. Cameron, Rannoch; and R. W. Meikle; Inverary. Seven candidates entered for the respective divisions and passed.

SECOND EXAMINATION.—Of forty-two candidates, the following twenty-seven passed: J. A. Featherstone, County Durham (with honours); W. H. Irwin, Leeds; C. E. Hargitt, Sheffield; J. Barry, County Cork; A. E. Clayton, South Shields; Margaret Penelope Monro, Edinburgh; F. J. Nolan, Dublin; J. Graham, Workington; Alexandra Mary Campbell Geddes, Bombay; Gertrude Keith, Edinburgh; Agnes Lillie Cousins, Madagascar; P. T. Finn, Ballymote; E. J. McCarthy Morris, County Cork; Sarah Brown McMordie, Belfast; A. W. Ball, Liverpool; C. G. Foggo, Morayshire; A. L. Levy, Melbourne; E. A. R. Laing, County Roscommon; Elizabeth Latto Ewan, Fyvie; M. McGann, County Clare; J. Moore, County Antrim; D. M. Atkinson, Yorkshire; A. Bance, Madras; A. P. Dias, Bombay; T. Mansour, Egypt; F. G. W. Deane, Barbadoes; and J. C. Forbes, Edinburgh. Of seventeen candidates who entered for the respective divisions ten passed.

FINAL EXAMINATION.—Of seventy-six candidates the following thirty-three passed, and were admitted L.R.C.P.E., L.R.C.S.E., and L.F.P. and S.G.: G. D. Robertson, Greenock; G. B. Thompson, Edinburgh; H. Keith, Ceylon; C. A. Courtney, Victoria; J. K. Browlees, Ballymena; C. S. Langley, County Cork: A. A. Macfarlane, Madras; T. Clegg, Oldham; W. C. Stevenson, Stirlingshire; M. à Beckett McCarthy, Sydney; C. W. J. Dunlop, County Down; H. H. Marshall, Madras; R. Williams, Saxilby; H. S. Smith, Toronto; P. McElwaine, Cavan; E. B. M. Atkins, Queenstown; W. H. Coupland, Liverpool; W. McKenzie Morison, Stornoway; A. J. Richards, Bangor; A. B. Franey, Banbury; F. F. Thorne, Kent; R. C. Macdonald, Arbroath; W. E. Wilson, Jamaica; D. Crichton, Edinburgh; K. Ganguli, India; W. E. Duckworth, India; J. Noonan, County Cork; H. D. Chapman, Staffordshire; F. C. Foster, New Zealand; W. Walker, Alford; A. J. Cromwell, Durham; C. G. Hoystead, Richmond; and C. S. Maunsell, Tramore. Of twenty-one candidates who entered for the respective divisions fourteen passed.

### EXAMINATION IN EDINBURGH FOR THE TRIPLE QUALIFICATION.

### PAPERS SET IN THE FIRST EXAMINATION.

QUESTIONS IN ELEMENTARY ANATOMY (all of which are to be answered).

1. Describe the Vertebral Column as a whole, and the Ligaments by which the Bodies and Laminæ of the true Vertebræ are connected.

2. Describe the Pectoralis Major Muscle in regard to its origin, insertion, relations, actions, nerve supply, and blood supply.

3. Describe the External or Musculo-Cutaneous Nerve of the Upper Extremity from its origin to its final distribution.

4. Name the branches of the Popliteal Artery in their order.

QUESTIONS IN CHEMISTRY (three of which are to be answered).

1. State by means of equations the preparation of the following substances: (*a*) Chlorine, (*b*) Iodine, (*c*) Hydrochloric Acid, (*d*) Hydriodic Acid. Explain the action of Chlorine as a bleaching agent.

2. What is meant by the oxidation and reduction of Salts? Illustrate your answer by means of equations, showing the action of Nitric Acid upon a Ferrous Salt, and the action of Sulphuretted Hydrogen on a Ferric Salt.

3. How is Hydrocyanic Acid prepared? What change does an aqueous solution of Hydrocyanic Acid undergo on keeping? What tests would you employ to detect the acid?

4. Olefiant Gas has the formula $C_2 H_4$. What is therefore its specific gravity—the specific gravity of air being = 1?

QUESTIONS IN PHYSICS (all of which are to be answered, and calculations shown where required).

1. Define the "Centre of Gravity" of a body. Show how to find, experimentally, the centre of gravity of an irregular lamina.

2. Prove that when an object is placed before a plane mirror its image is seen as far behind the mirror as the object itself is before it. Wherein does the image differ from the object?

3. What are meant by saying (1) that the Specific Heat of copper is ·095; and (2) that the Latent Heat of water is 80 units?

4. How would you magnetise a bar of soft iron by means of a voltaic current? How would you alter the polarity of the bar?

The Hospital Advertiser.

